# The variability of antennal sensilla in Naucoridae (Heteroptera: Nepomorpha)

**DOI:** 10.1038/s41598-021-99067-5

**Published:** 2021-10-04

**Authors:** Agnieszka Nowińska, Jolanta Brożek

**Affiliations:** Faculty of Natural Science, Institute of Biology, Biotechnology and Environmental Protection, University of Silesia in Katowice, Bankowa 9, 40-007 Katowice, Poland

**Keywords:** Entomology, Scanning electron microscopy

## Abstract

The morphology and distribution of sensilla on the surface of the antennae of the naucorids’ species were studied via scanning electron microscopy. Eleven types of sensilla were identified regarding specific sensory modalities, based on their cuticular morphology. Cuticle morphology identifies five types of sensilla trichodea, four types of sensilla basiconica, one type of sensillum coeloconicum and sensillum ampullaceum. Three new types of mechanosensitive sensilla were found. Moreover, the morphological diversity between the antennae allowed the distinction of ten different antennal types that correspond to different sensillar sets. The sensilla found in Naucoridae share similarities with the sensilla of other nepomorphan taxa, as well as of terrestrial insects. However, no sensillar synapomorphy was found between Naucoridae and Aphelocheiridae.

## Introduction

The insects' fundamental receptive organ is the sensillum (commonly called sensory hair, sensory peg, sense organ, or sensory receptor). They are the structures, on the insects' body, responsible for receiving signals from the environment^1–3^. Sensilla are distributed on a range of insect structures, including the antenna, mouthparts, and limbs, with emphasis on the tarsi^4^. As external cuticular parts are the most accessible for study, there are many detailed studies, particularly about the pores and shapes of sensilla, as well as the type of connection with the cuticle (socked flexible or inflexible). According to their cuticular structure and embedding in the cuticle, sensilla are divided into aporous sensilla with flexible or inflexible sockets, and porous sensilla (always without flexible sockets). Moreover, the functional categories of sensilla (mechano-, chemo-, thermo- and hygroreceptive) have been supported by ultrastructural studies on the receptors (number of neurons, branched or not branched dendrites, and sheath cells)^5–9^.

Touch, physical pressure, movement, stretching, vibrations, and contractions all serve to alter the position of the cuticle of the mechanosensilla. This, in turn, transmits the stimulus through the dendritic sheath to the membrane of the tubular body deformation, which initiates electrical activity^3^. Mechanoreceptors perceive external stimuli during certain behavioural activities such as locomotion, posture, feeding, orientation, oviposition, and hearing. Moreover, one type of sensillum may be responsible for more than one function. In insects, these structures usually bear no pores and are innervated by a single neuron. Mechanoreceptors usually bear a membrane, connecting the body of the sensilla with the cuticle of the antenna, allowing greater mobility at the base. The common types of mechanoreceptors are sensilla trichodea, sensilla chaetica and sensilla campaniformia^2,9,10^.

Chemoreceptors may consist of one or more sensory neurons and are responsible for two main functions: olfaction and gustation. Zacharuk^11^ distinguishes two types of chemoreceptive sensilla. The first type is the uniporous sensillum, which has one opening in the cuticle, allowing chemical communication. These structures usually sense via contact or gustation. However, they can also respond to odour. The second type of chemoreceptive organ is the multiporous sensillum. In comparison to uniporous sensilla, they have a relatively larger number of pores on the surface and are mainly responsible for olfaction. Their sockets are inflexible, which means that in contrast to mechanoreceptors, they do not have a membrane connecting the body of the sensillum with the cuticle of the antenna. The common types of chemoreceptive sensilla are sensilla basiconica, sensilla coeloconica, sensilla trichodea and sensilla placodea^9,11^.

Thermo-hygroreceptors are still poorly known in comparison to other sensors. Their general role is controlling water balance and sense humidity and temperature variations^12^. A typical thermo-hygroreceptive sensillum contains 2–5 sensory cells (commonly three)^6^, and as well as the chemoreceptive sensilla, they are embedded in an inflexible socket. The common types of thermo-hygroreceptive sensilla are sensilla coeloconica and sensilla ampullacea^9^.

Although the whole insect body is covered with sensilla, the majority of different sensillum types responsible for receiving different signals are found on the antennae, particularly the olfactory sensilla^4,6^. The antennae are the subject of interest in this study, we analyse if the olfactory sensors are morphologically changed for water habitats and if the habitat have influenced the mechanosensilla’s shape in naucorid species.

Naucoridae is divided into five subfamilies and 34 genera and are distributed worldwide^13,14^. They have a short rostrum and a short, four-segmented antennae^13,14^. They mostly live in streams, however species frequent different habitats among subfamilies. According to Chen et al.^13^, the species of the subfamily Cheirochelinae prefer water areas with a strong current, whereas species of Laccocorinae live in streams, staying at the edges or in quiet pools and avoid strong currents. Most of the species of Naucorinae occur in stagnant water or sluggish bays of streams. Parsons and Hewson^15^ described species of Cryphocricinae living in rapid streams under stones and gravel; Rodrigues and Sites^16^ stated species of Limnocorinae usually occur in shallow streams, and that they are associated with leaf packs or roots of riparian vegetation. There is a small amount of data about the naucorids' feeding habits. As stated by Hungerford^17^ they mainly feed on insects, although Poisson^18^, Sites^19^ and Wade et al.^20^ also mentioned aquatic snails, small crustaceans, and fish; and there is one record of necrophagy in *Naucoris maculatus* Fabricius, 1798^21^. Naucorid species are good swimmers, however species of Naucorinae usually wait between the plants for the prey to come close^13^. La Rivers^22^ described both swimming and crawling for the genus *Ambrysus* Stål, 1862, depending on the habitat; according to this author, these insects have poor sight and only react to movement if it happens at less than two times their body-length distance.

Data on antennal morphology, and particularly antennal sensilla in Naucoridae is very scarce. Antennal sensilla in Nepomorpha, in a general term, were mentioned by Popov^23^, Schuh and Weirauch^14^ and Chen et al.^13^. Some data regarding the type sensilla in one Naucorid species—*Ilyocoris cimicoides* (Linnaeus, 1758) was analyzed by Sinitsina and Chaika^24^.

The relationships inside the superfamily Naucoroidea are divergent based on previous different morphological and molecular data sets. Štys and Jansson^25^ and Ye et al.^26^ regarded Naucoroidea as a clade that included Naucoridae + Aphelocheiridae + Potamocoridae. China^27,28^ distinguished Naucoroidea (as Naucoridae + Aphelocheiridae), but Popov^23^ and Mahner^29^ found a relationship between the Potamocoridae and the clade Naucoridae + Aphelocheiridae. Hebsgaard et al.^30^ presented Naucoroidea with a single family (Naucoridae), while Rieger^31^ and Brożek^32^ considered Naucoroidea (Naucoridae + Potamocoridae).

In this study, we characterize and determine the abundance and distribution of the antennal sensilla of these nepomorphan bugs using scanning electron microscopy (SEM), we compare antennal sensilla among the subfamilies of Naucoridae with emphasis on their adaptations to different habitats, and we compare our results with other nepomorphan families^24,33–35^, in particular with Aphelocheiridae.

## Materials and methods

The antennal sensilla of eleven species from five subfamilies of Naucoridae were studied with the use of a scanning electron microscope, 1–4 adult specimens of each species were used. Sexual dimorphism has not been taken into consideration.

All the specimens have been cleaned in an ultrasonic cleaner and dried in ethanol. Next, they were placed on stubs, coated with gold or chromium, and analysed with the use of a Phenom XL and Hitachi UHR FE-SEM SU 8010 scanning electron microscope in the scanning microscopy laboratory of the Faculty of Biology and Environmental Protection of Silesian University in Katowice. Most of the material was obtained thanks to the donation of Ping-ping Chen (private collection). The rest belong to the collections of the National Museum in Prague, Hungarian Natural History Museum in Budapest, and the Zoological Museum of the State Moscow University.

The following species have been examined.

**Cheirochelinae**: *Asthenocoris australis* Zettel, Nieser & Polhemus, 1999 (Philippines, Mindanao, N9326).
  *Gestroiella limnocoroides* Montandon, 1897 (Indo-China & Annam Laos).
**Cryphocricinae**: *Ambrysus fuscus* Usinger, 1946 (Real de Arriba Temescaltepec Mex.)
  *Cataractocoris marginiventris* Usinger, 1941 (Temescaltepec Mex.)
  *Cryphocricos montei* De Carlo, 1951 (Brazil, Minas Gerais).
**Laccocorinae**: *Heleocoris strabus* Montandon, 1897 (Thailand, Chiang Rai).
  *Laccocoris* sp. Stål, 1856 (Bukoba Victoria Nyanza Troitzkij).
**Limnocorinae**: *Limnocoris asper* Nieser & López Ruf, 2001 (Brazil, Minas Gerais).
  *Limnocoris pusillus* Montandon, 1897 (Brazil, Minas Gerais).
  *Limnocoris volxemi* Lethierry, 1877 (Brazil, Minas Gerais).
**Naucorinae**: *Naucoris cimicoides* Linnaeus, 1758 (Balaton & Hoštice, Sil. ČSR.2.10.1934).
  *Naucoris scutellaris* Stål, 1860 (Thailand, Mae Hong Son).
  *Pelocoris femoratus* Palisot, 1820 (U.S.A., Illinois).
  *Pelocoris poeyi* Guérin-Méneville, 1838 (Curaçao).

## Results

### General morphology of antennae

Antennae usually consist of four antennomeres—scape (I), pedicel (II), and two flagellomeres (III and IV)—that are relatively short (around 500 µm) and wide but, in some species, are slightly longer (up to 1100 µm) (Table 1). In the present study, only antennomeres from II–IV are shown in *Ambrysus fuscus*, *Cataractocoris marginiventris* and *Laccocoris* sp., because the scape (antennomere I) was destroyed during dissection. In the remaining studied species, a particular variation in the antennomeres' size and shape was observed and various types of antennae were recognized within subfamilies.

**Table 1:** The data of the size of some antennomeres in studied species.

Species	Approximate length of the antenna (µm)	Approximate width of the antenna	
		Thinnest antennomere (number—width in µm)	Widest antennomere (number—width in µm)
*Asthenocoris australis*	630	IV—50	I—100
*Gestroiella limnocoroides*	750	III—123	I—185
*Ambrysus fuscus*	723	IV—67	II—148
*Cataractocoris marginiventris*	Scapus damaged	IV—66	II—162
*Cryphocricos montei*	615	III—43	I—95
*Heleocoris strabus*	733	IV—57	II—166
*Laccocoris* sp.	Scapus damaged	IV—65	II—214
*Limnocoris asper*	833	IV—55	II—106
*Limnocoris pusillus*	489	III—40	II—84
*Limnocoris volxemi*	827	IV—55	II—250
*Naucoris scutellaris*	500	IV—54	II—110
*Naucoris cimicoides*	1188	IV—85	II—255
*Pelocoris femoratus*	716	IV—65	II—186
*Pelocoris poeyi*	673	IV—70	II—196

**Figure 1:**
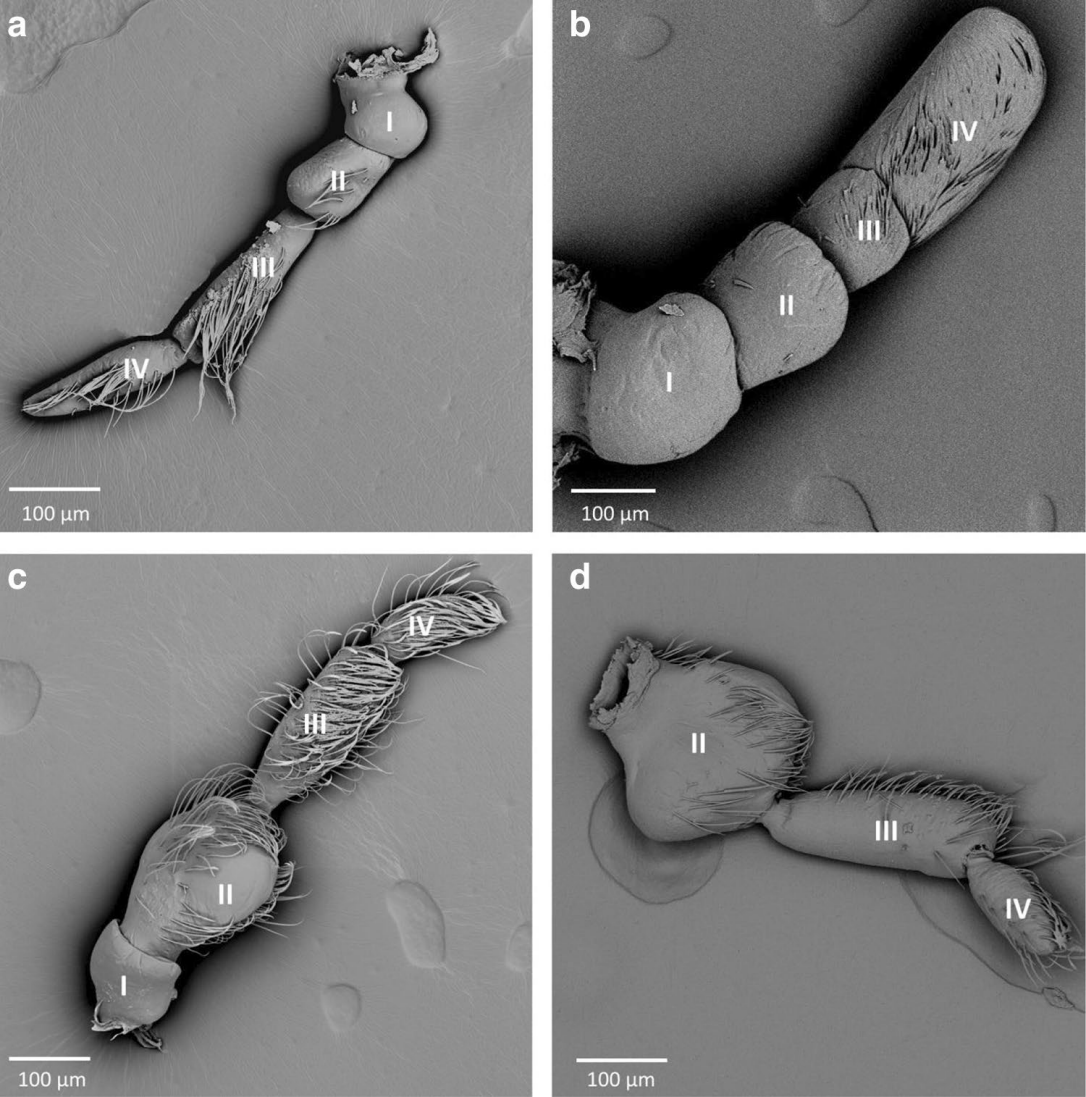
The antennae of Cheirochelinae and Laccocorinae; (**a**) *Asthenocoris austrialis*, (**b**) *Gestroiella limnocoroides*, (**c**) *Heleocoris strabus*, (**d**) *Laccocoris* sp.; I–IV—numbers of antennomeres.

In Cheirochelinae, two types of antennae are visible; the first was observed in *Asthenocoris australis* (Fig. 1a), and the second was characteristic of *Gestroiella limnocoroides* (Fig. 1b). In *A. australis,* the scape and pedicel are similar in size (short) and shape (slightly trapezoid); the first flagellomere is the longest and slightly slender than the scape and pedicel. The second flagellomere is shorter than the first one, but longer than scape and pedicel, and is apically acute. In *Gestroiella limnocoroides,* all antennomeres have almost the same width; the first two are very similar in length (short) and shape (a flattened trapezoid), the third is straight and the shortest. The last one is the longest and blunt ended.

**Figure 2:**
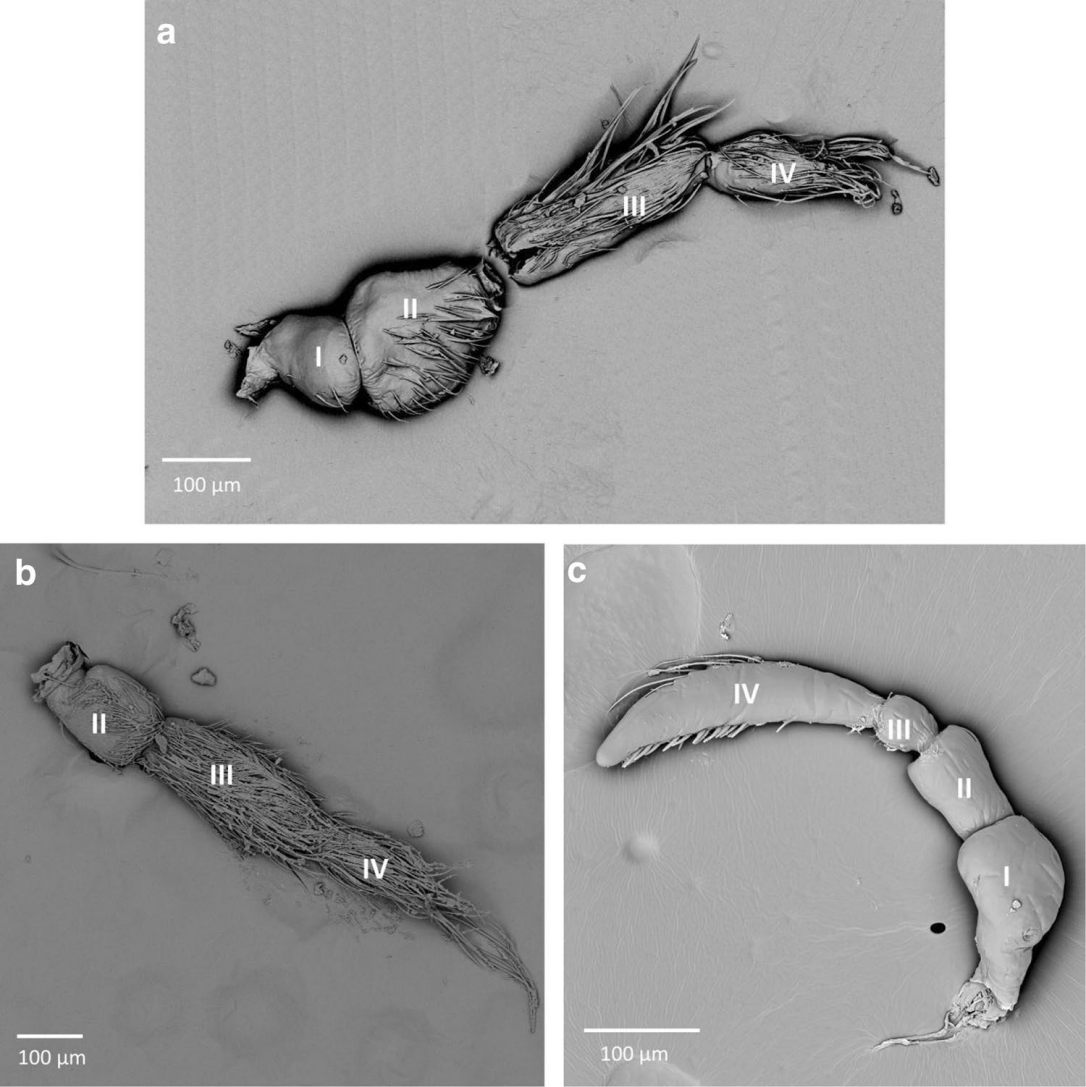
The antennae of Cryphocricinae; (**a**) *Ambrysus fuscus*, (**b**) *Cataractocoris marginiventris*, (**c**) *Cryphocricos montei*; I–IV—numbers of antennomeres.

Three types were found in Cryphocricinae; in *Cryphocricos montei* (Fig. 2c), the scapus is wide and of medium length. The first flagellomere is the shortest and narrow but the second flagellomere is wide and the longest, with a crescent-like shape. These features differentiate the antennae of *Cryphocricos* from the other two types of antennae observed in the subfamily. In *Ambrysus fuscus* (Fig. 2a)*,* we observe a difference in the longest and widest antennomere: the third antennomere is the longest and the second is the widest, and the fourth antennomere is the shortest. In *Cataractocoris marginiventris* (Fig. 2b), due to the destruction of the scape, its shape and size cannot be described. Antennomeres 2–4 are more or less the same width, narrowing slightly from the base of antennae to the apex. The third antennomere is the longest and the fourth is the shortest. The general shape of the antennae of *Cataractocoris marginiventris* resembles the antennae of *Astenocoris austrialis*.

Another type of antennae was characteristic for Laccocorinae. In *Heleocoris strabus* (Fig. 1c), the scapus is small and narrow compared to the pedicel. In both *Heleocoris strabus* and *Laccocoris* sp. (Fig. 1c,d) the pedicel is the widest and possesses a visible lateral protuberance. The first flagellomere is the longest and quite wide, whereas the second flagellomere is significantly shorter and slightly narrow. This shape of the antennae resembles the one of *Ambrysus fuscus*.

**Figure 3:**
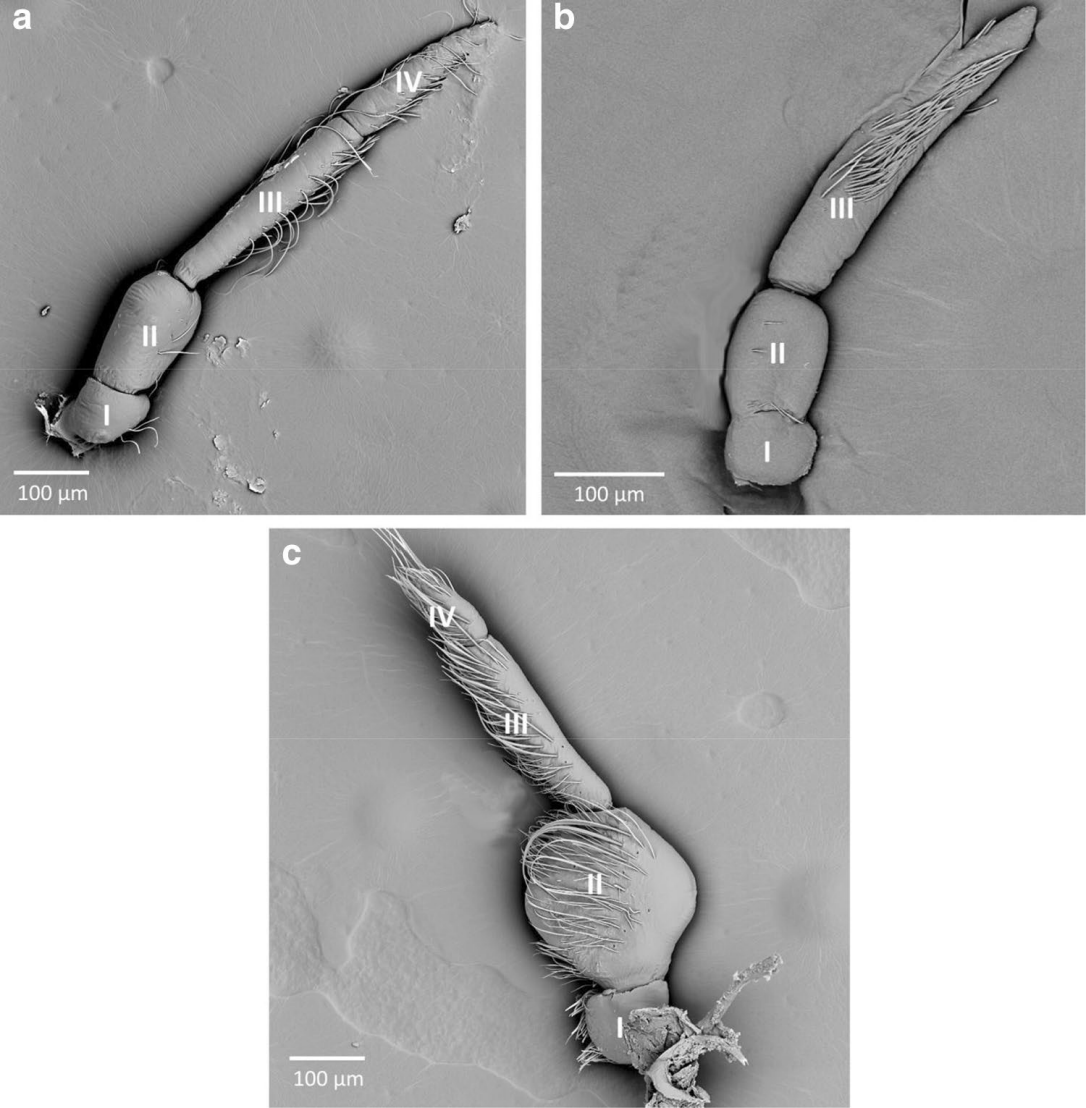
The antennae of Limnocorinae; (**a**) *Limnocoris asper*, (**b**) *Limnocoris pusillus*, (**c**) *Limnocoris volxemi*; I–IV—numbers of antennomeres.

Three types of antennae were recognized in Limnocorinae; in *Limnocoris asper* (Fig. 3a) the scapus is short and narrow, the pedicel is the widest, the first and second flagellomeres are longer and narrow. The first flagellomere is the longest, whereas the second is of medium length and pointed apically. The boundaries between flagellomeres are slightly visible. *Limnocoris pusillus* (Fig. 3b) possess only one long, pointed flagellomere; the scapus and pedicel are the same as in *L. asper*. In *Limnocoris volxemi* (Fig. 3c), the pedicel has a visible protuberance; the first flagellomere is long and narrow, but the second is very short and thin. The described features make the antennae of *Limnocoris volxemi* resemble the ones from *Ambrysus fuscus* and Laccocorinae.

**Figure 4:**
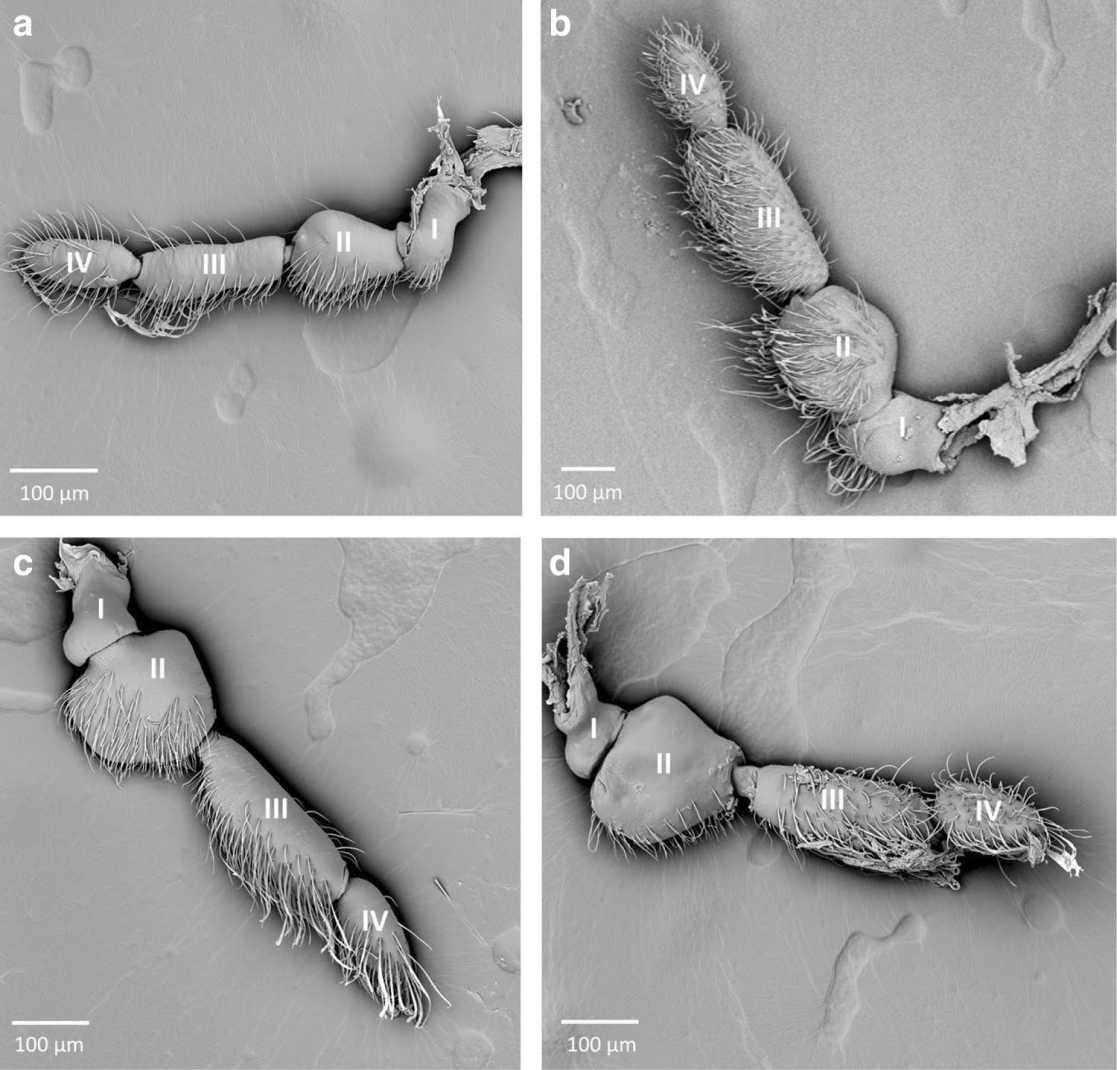
The antennae of Naucorinae; (**a**) *Naucoris scutellaris*, (**b**) *Naucoris cimicoides*, (**c**) *Pelocoris femoratus*, (**d**) *Pelocoris poeyi*; I–IV—numbers of antennomeres.

One type of antennae was distinguished in Naucorinae; all tested species (*Naucoris cimicoides, Naucoris scutellaris, Pelocoris femoratus and Pelocoris poeyi*) (Fig. 4) present substantial morphological similarities when it comes to the antennomeres. The scapus and pedicel at the base are the same width; the pedicel is slightly longer and possesses a broad lobate protuberance. The first and second flagellomeres are the same width, but the second is significantly shorter than the first one and has a rounded tip.

### Typology, morphology, and distribution of sensilla

In total, five main types of sensilla were found on the antennae, including sensilla trichodea (ST), sensilla campaniformia (SCa), sensilla basiconica (SB), sensilla coeloconica (SCo) and sensilla ampullacea (SA). We follow the terminology and classification of sensilla reported in other papers^6,33–35^. The detailed distributions of the sensilla in each of the studied species are presented in Table 2.

**Table 2:**
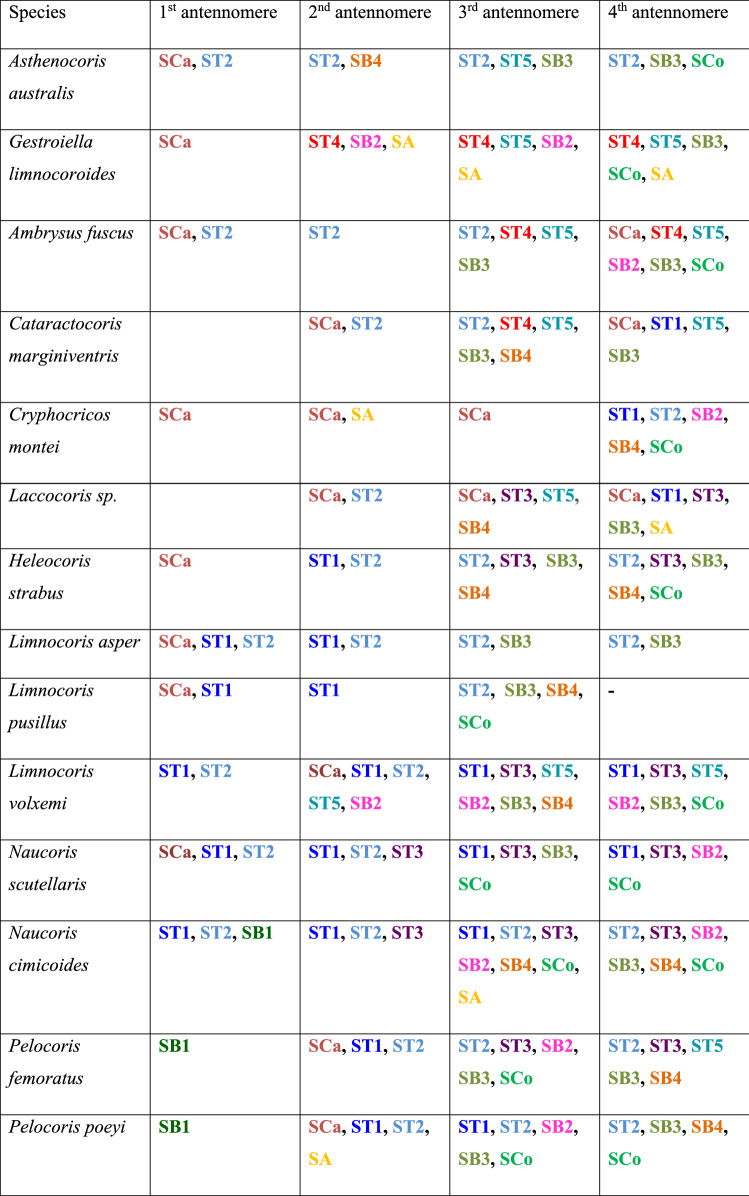
Distribution of sensilla types on the antennomeres (the colours correspond to the ones used in Figs. 5, 6, 7, 8 and 9).

**Figure 5:**
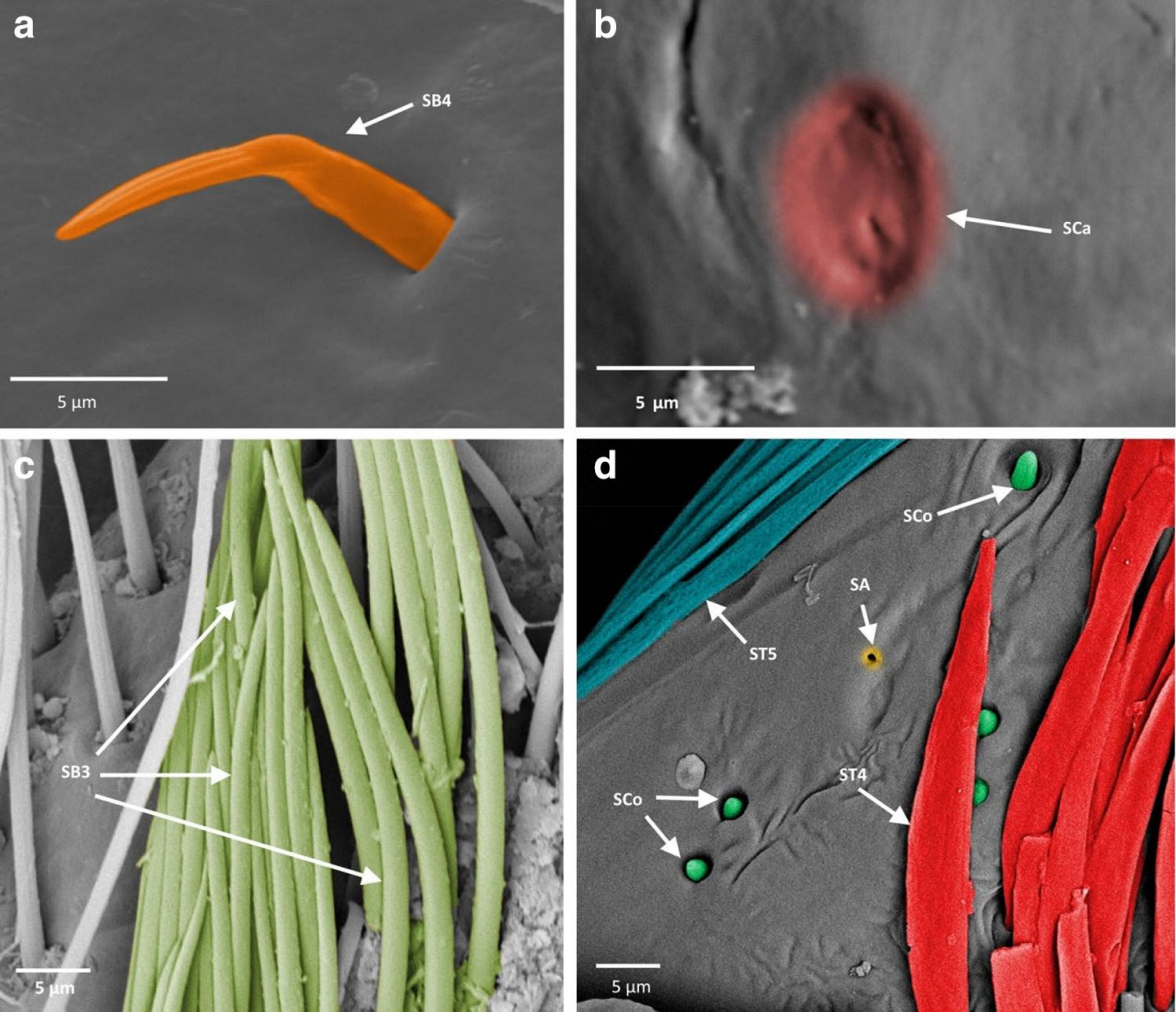
Antennal sensilla; (**a**–**c**) *Asthenocoris austrialis*, (**d**) *Gestroiella limnocoroides*. *ST* sensilla trichodea, *SCa* sensilla campaniformia, *SB* sensilla basiconica, *SCo* sensilla coeloconica, *SA* sensilla ampullacea.

**Figure 6:**
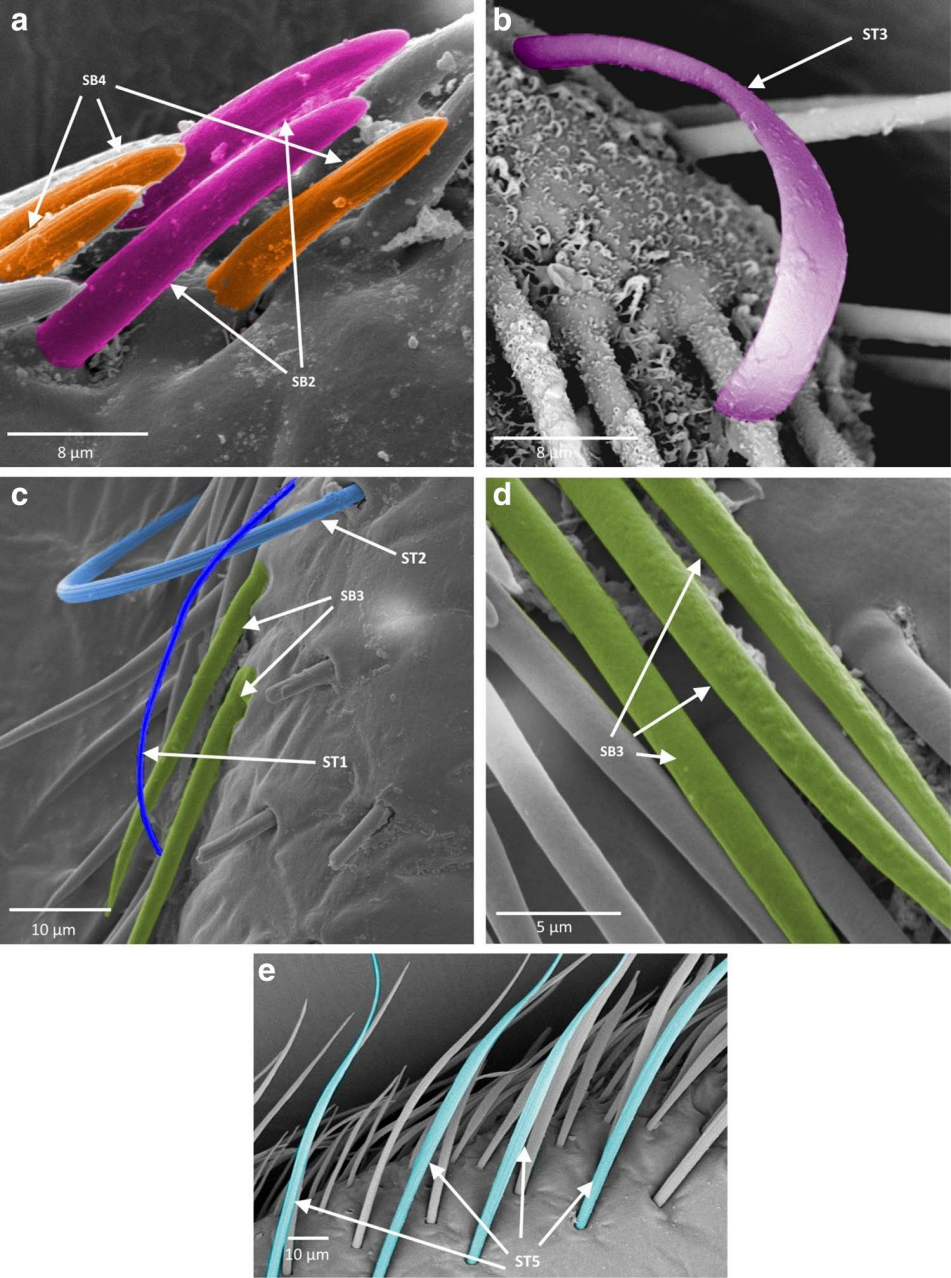
Antennal sensilla; (**a**)* Cryphocricos montei,* (**b**)* Heleocoris strabus,* (**c**)* Limnocoris asper,* (**d**)* Limnocoris pusillus,* (**e**)* Limnocoris volxemi*. *ST* sensilla trichodea, *SB* sensilla basiconica.

**Figure 7:**
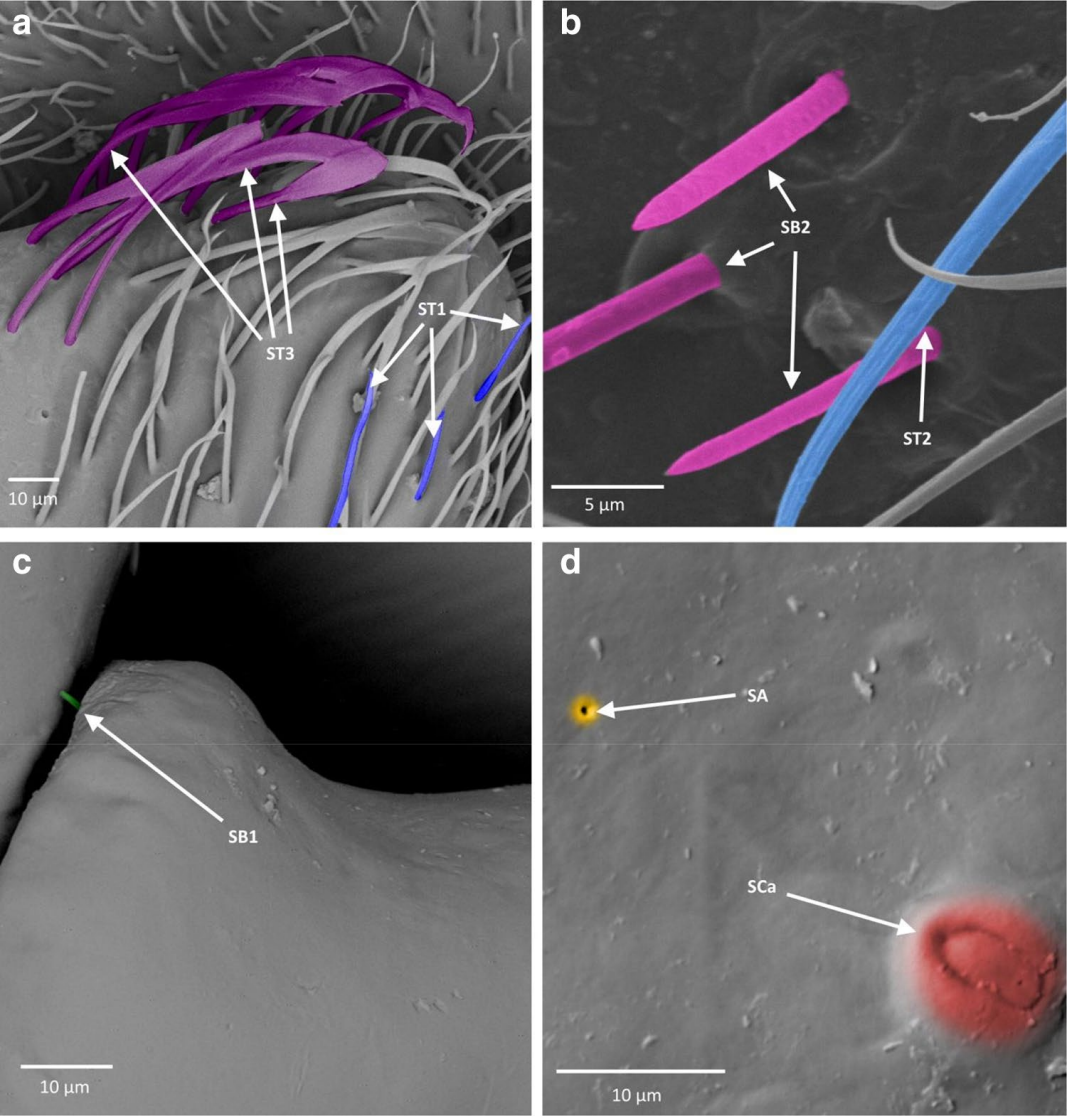
Antennal sensilla; (**a**)* Naucoris scutellaris*, (**b**)* Naucoris cimicoides*, (**c**) *Pelocoris femoratus*, (**d**)* Pelocoris poeyi*. *ST* sensilla trichodea, *SCa* sensilla campaniformia, *SB* sensilla basiconica*.*

**Figure 8:**
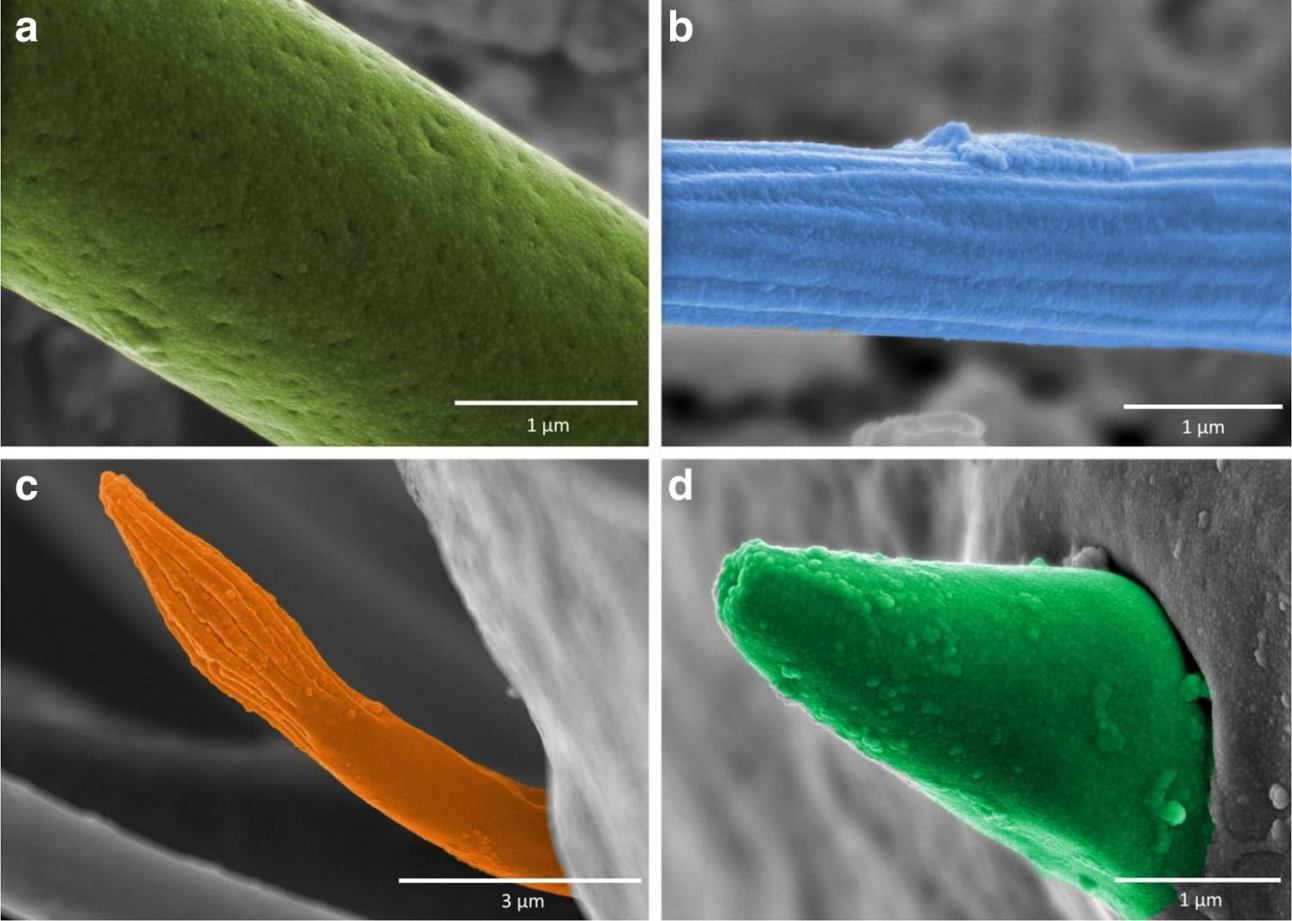
Antennal sensilla; (**a**) the surface of sensilla basiconica SB3 in *Cataractocoris marginiventris*, (**b**) the surface of sensilla trichodea ST2 in *Cataractocoris marginiventris*, (**c**) sensilla basiconica SB4 in *Laccocoris* sp., (**d**) sensilla coeloconica SCo in *Laccocoris sp.*

**Figure 9:**
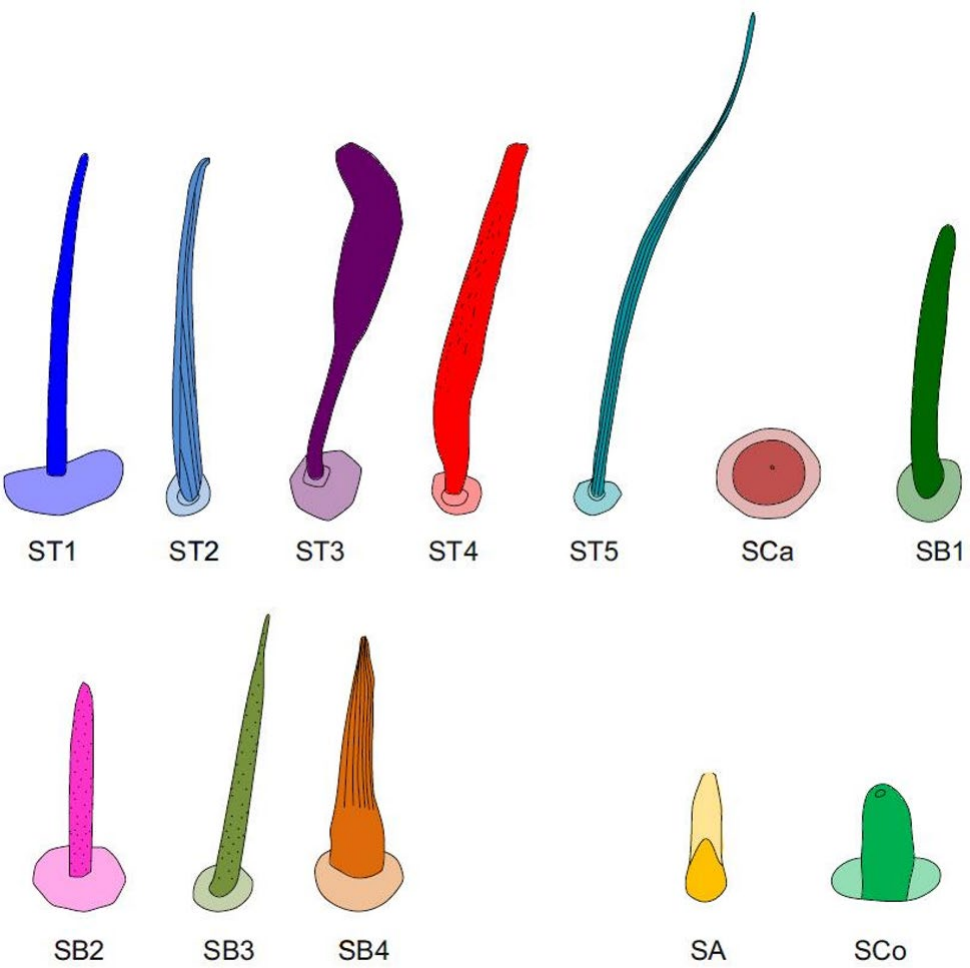
The schematics of antennal sensilla of Naucoridae. *ST* sensilla trichodea, *SCa* sensilla campaniformia, *SB* sensilla basiconica, *SA* sensilla ampullacea, *SCo* sensilla coeloconica.

#### Sensilla trichodea

These were the most numerous and the longest type of sensilla in the studied species. They can be divided into five subtypes (ST 1–5), according to different morphological characters (change from the oval trichoid shape to more flattened), different microstructures of the sensillar wall, and length.

##### Sensilla trichodea ()

Long, straight, hair-like sensilla, with a smooth surface, pointed tip and a flexible socket. These sensilla were found in different quantities, on all the antennomeres in all the species except in the subfamily Cheirochelinae (Figs. 6c, 7a, 9, Table 2).

##### Sensilla trichodea ()

Long, straight, hair-like sensilla, with a ribbed surface, pointed tip and a flexible socket. The ST2 is the most common type of sensilla trichodea, it was found on all the antennomeres and occurred in all the studied species except *Gestroiella limnocoroides* (Figs. 6c, 7b, 8b, 9 Table 2).

##### Sensilla trichodea ()

Long, leaf-like shaped sensilla, flattened, with a comparatively thinner base and a smooth or slightly ribbed surface. Embedded in a flexible socket. These sensilla usually occurred on the last two antennomeres of *Heleocoris strabus*, *Laccocoris* sp., *Limnocoris volxemi, Naucoris scutellaris*, *Naucoris cimicoides* and *Pelocoris femoratus* (Figs. 6b, 7a, 9, Table 2).

##### Sensilla trichodea ()

Long, flattened sensilla, with irregular microsculptures on the surface. The shaft is more or less the same width along the whole length, ending in a round tip. Embedded in a flexible socket. This type of sensillum was present in *Gestroiella limnocoroides*, *Ambrysus fuscus* and *Cataractocoris marginiventris* on the second, third and/or fourth antennomeres (Figs. 5d, 9, Table 2).

##### Sensilla trichodea ()

The longest sensilla, slightly flattened and ribbed, with more or less the same width throughout most of the length, with a long thin tip. Embedded in a flexible socket. This type of sensillum was found only in *Asthenocoris austrialis*, *Gestroiella limnocoroides*, *Ambrysus fuscus*, *Cataractocoris marginiventris*, *Laccocoris* sp., *Limnocoris volxemi* and *Pelocoris femoratus*, on the second, third and fourth antennomeres (Figs. 5d, 6e, 9, Table 2).

#### Sensilla campaniformia ()

The typical shape was observed. It is a flat and round sensilla, with a pore in the middle (molting pore) and a flexible socket. It is a common type of sensilla for insects and occurred in all studied species on different antennomeres (Figs. 5b, 7d, 9, Table 2).

#### Sensilla basiconica

One aporous mechanoreceptive type (SB1) and three porous chemoreceptive types (SB2–SB4) were observed. Such sensilla are a common olfactory sensilla in insects.

##### Sensilla basiconica ()

Long, straight cones, without pores on the surface, embedded in a flexible socket. They occur as individual structures between two antennomeres and work as a proprioceptive sensilla. These sensilla basiconica were documented in *Naucoris cimicoides*, *Pelocoris femoratus* and *Pelocoris poeyi*. This proprioceptive sensillum is common in insects (Figs. 7c, 9, Table 2).

##### Sensilla basiconica ()

Short cones with a round or flattened cross-section. With pores on the whole surface, embedded in an inflexible socket. This type of sensillum occurred on the second, third and/or fourth antennomere in *Gestroiella limnocoroides*, *Ambrysus fuscus*, *Cryphocricos montei*, *Limnocoris volxemi* and in the studied species of the subfamily Naucorinae (Figs. 6a, 7b, 9, Table 2).

##### Sensilla basiconica ()

Long structures, flattened with a thicker base and a thinner, pointed tip. Porous on the whole surface. Embedded in an inflexible socket. These sensilla occurred on the third and fourth antennomeres in all the species, except *Cryphocricos montei* (Figs. 5c, 6c,d, 8a, 9, Table 2).

##### Sensilla basiconica ()

Short cones, thicker at the base and thinner at the top. The base of these sensilla is smooth and the rest ribbed and porous. Embedded in an inflexible socket. This type of sensillum was observed in *Asthenocoris austrialis*, *Cataractocoris marginiventris*, *Laccocoris* sp., *Cryphocricos montei*, *Heleocoris strabus*, *Limnocoris pusillus*, *Naucoris cimicoides* and *Pelocoris* on the second, third and/or fourth antennomeres (Figs. 5a, 6a, 8c, 9, Table 2).

#### Sensilla coeloconica ()

Short pegs embedded in an inflexible socket with a pore on the surface. This sensillum was found on the dorsal surfaces of the third and/or fourth antennomeres and on the tip of the last one. They were documented in all the studied species except *Cataractocoris marginiventris*, *Laccocoris* sp. and *Limnocoris asper* (Figs. 5d, 8d, 9, Table 2).

#### Sensilla ampullacea ()

Pegs embedded below the surface of the cuticle, with a round opening visible from the surface of the antennae. Ultrastructural studies need to be done to confirm this type of sensillum since we are not able to see its surface (nonetheless, their surface probably is aporous). In this study, they were found in *Gestroiella limnocoroides*, *Cryphocricos montei*, *Laccocoris* sp., *Naucoris cimicoides* and *Pelocoris poeyi* on the second, third and fourth antennomeres (Figs. 5d, 7d, 9, Table 2).

## Discussion

The present study is the first to characterize the antennal sensillar equipment and the antennal morphology across all the subfamilies of Naucoride and covering the entire lineage using an electron microscope observation.

This represents a great advance since, as far as we know, the only studies on the antennal sensory structures of Naucoridae covered only one species—*Ilyocoris comicoides*^24^.

### Variability of shapes of the antennae

Irrespective of any degree of segmentation of the antenna, the scape, the pedicel, and the flagellum, are remarkably conserved throughout the insects. The antennae of most heteropteran infraorders are quite uninformative: throughout the whole post-embryonic development, from the first nymphal stage to the adult, the number of antennomeres is uniformly four^36^. Nevertheless, this conservation is probably not universal, because some differentiation is seen in some insects^36^. Most studied taxa of Naucoridae, despite having four segmented antennae, display various morphological shapes and sizes of antennomeres (Table 1). However, we found one case of three-segmented antennae in *Limnocoris pusillus*. Moreover, the morphological variability is visible between subfamilies and is even noted between the species of the same genera of *Limnocoris*, as well as between species of Cheirochelinae and Cryphocricinae. In comparison to other nepomorphan families, the antennae of the Naucoridae are the most diverse, and various types of antennae can be found within the subfamilies. In other families, the subfamilies or at least the genera are usually characterized by a similar shape of antennae^33–35^. A possible process of oligomerization of the third and fourth antennomeres was observed in the *Limnocoris pusillus*. Generally, the antennae of Naucorid species are short in comparison to the elongated antennae in Aphelocheiridae and Ochteridae^13,23^. The other process of morphological specialization in heteropteran taxa led to the proliferation of five segmented antennae in Pentatomomorpha^14^.

### Antennal sensory equipment in Naucoridae

#### Mechanosensilla

As in other nepomorphans, and insects in general, a few common sensilla types can be assigned to each subfamily of naucorids. The morphology and function of mechanoreceptive sensilla in the studied species are classified into three basal types: sensilla trichodea, sensilla campaniformia and sensilla basiconica^4,6^. However, we observed unique shapes of sensilla trichodea (ST1–ST5) in some subfamilies.

Cheirochelinae species (*Asthenocoris australis*) display sensilla trichodea ST2 on all antennomeres and ST5 on the third antennomere. In *Gestroiella limnocoroides* however, which lacks the ST2, sensilla ST4 and ST5 are more abundant on the second and fourth antennomeres. Cheirochelinae species lack the ST1. A curious case of sensilla arrangement was observed in the Cryphocrycinae: ST4 and ST5 are present on the third and fourth antennomeres in *Ambrysus fuscus* and *Cataractocoris marginiventris* but not in *Cryphocricos montei*. Another type of arrangement of sensilla trichodea (ST1, ST2) occurs in the aforementioned species: in *Cryphocricos montei,* ST1 and ST2 are only present in the last antennomere, while in *Cataractocoris marginiventris* ST2 are present on the second and third antennomeres and ST1 on the last antennomere. Moreover, *Ambrysus fuscus* lacks ST1, but ST2 are present on antennomeres 1–3.

Although ST1 and ST2 are common types of sensilla in naucorids (Table 2) and other nepomorphans^33–35^, their configuration of having only ST1 and ST2 on the last antennomere, could be treated as a unique character (autapomorphy?) of *Cryphocricos montei*. The species possesses only these trichoid mechanosensilla.

In the Laccocorinae (*Heleocoris strabus*), many sensilla trichodea (ST1, ST2, ST3) were found on the antennomeres 2–4, but in *Laccocoris* sp. ST5 sensilla were also present, like what is found in some species of Cheirochelinae and Cryphocricinae. The Limnocorinae species displayed sensilla trichodea (ST1 and ST2) on all four antennomeres, except in the case of *Limnocoris pusillus*. The latter possesses only three antennomeres, the first two with ST1 and the last with ST2. A peculiar phenomenon was the appearance of more sensilla types (ST1, ST2, ST3, and ST5) in *Limnocoris volxemi* than in the other species of the same genus. In Naucorinae, a pattern for the arrangement of ST1, ST2, and ST3 sensilla, was specific to individual taxa. For the two species of *Naucoris*, the distribution pattern was almost identical. In two species of *Pelocoris*, the arrangement of sensilla ST1, ST2, and ST3 did not differ substantially. In comparison to *Naucoris*, the first antennomere in *Pelocoris* was devoid of sensilla ST1 and ST2. Nevertheless, in *Pelocoris femoratus*, ST5 were also present on the last antennomere, in addition to ST1, ST2 and ST3. In summary, the mechanosensillum ST4 was found in some of Cheirochelinae and Cryphocricinae species, whereas ST3 were present only in some Laccocorinae, Limnocorinae and Naucorinae species.

In contrast with other nepomorphans previously studied, regarding their shapes, the sensillum ST3, ST4 and additional ST5 found in Naucoridae are new types of mechanosensilla. Nepoidea's sensillum ST3 and Corixioidea’s sensilla ST3 and ST4 are straight, thick structures with a rounded or pointed tip and ribbed surface^33–35^, and are different than the sensilla described in the present study as ST3 and ST4: Cheirochelinae species show a long, straight sensilla, flattened and with almost the same width through the whole length. On the other hand, ST1 and ST2 sensilla as described in Ochteridae, Gelastocoridae and Aphelocheiridae are identical to naucorid species^34^. The five different types of trichoid mechanosensilla from the studied species of Naucoridae represent different methods of gathering mechanical information from the environment. Most of the species studied possess the basal type ST1 and ST2. Sensilla trichodea's density varies from low to high without an apparent relation on the taxonomic variation of the subfamilies.

Sensilla trichodea were also observed in all pentatomid species^37^ and are like the ST1 in naucorids; and ST1 and ST2 sensilla found in Tingidae's^38^ are identical in naucorid species. Mechanosensilla chaetica types are absent in Naucoridae, although they are present in many other Heteroptera^33,34,37,39,40^.

When looking at the mechanosensilla complement of the antennae of Naucoridae, we also observe the presence of sensilla campaniformia (SCa) and proprioceptive sensilla basiconica (SB1), the latter located proximally in the dorsal side of the antennomeres. Both types of sensilla have specific specializations towards tension of the cuticular structures. Their arrangements and shapes are similar for all nepomorphans^33–35^ and probably for broader groups of heteropteran species^38,41^.

Mechanoreception is widely used by many organisms and is one of the essential sense organs used by different water organisms (e.g. pelagic and benthic crustaceans and water insects) in the detection of predators, prey and mates, as well as communication. Behavioural studies, especially in crustaceans, have demonstrated that mechanosensilla affect the response, whether to attack, escape from, or pursue the source of the stimulus. However, the water environment places constraints on the receptors' design due to the physics of inhabiting a viscous environment with no fixed reference point. Thus, near-field disturbances are detected as differences in water flow between the sensor and the organism. In insects, these hydro-mechanical cues are transduced into a biological signal by cuticular sensilla to inner mechanoreceptor cells; crustaceans have scolopidial mechanoreceptors. Cuticular sensilla respond to tactile cues, water flow, or bending. The basic mechanisms appear to be similar to those in benthic crustaceans and insects^42^. Probably, the morphological differences, especially the flattened types of mechanosensilla (ST3, ST5, ST5) and significant variation in the number of the mechanosensilla in the naucorids relate to the water lifestyle. In Nepoidea and Corixoidea we also found the typical and flattened mechanosensilla^33,35^. It is also known that the ST1, ST2, SCa and SB1 are widespread in terrestrial insects. The main thing to keep in mind is that nepomorphans are secondary aquatic organisms^23,27^, so some adaptations of the mechanosensilla to the environment are evident.

#### Chemo/olfactory and thermo-hygrosensilla

The olfactory system shows, as a rule, a wide range of morphological diversity among different insect’s taxa^11^; however, the olfactory sensilla are frequently very similar^43^. The antennal sensilla of Naucoridae show specialization towards olfaction, with a diversification into three subtypes of sensilla basiconica (SB2, SB3, SB4). However, the sets of olfactory sensilla are different in some species (Table 2). According to Slifer’s^5^, and Altner and Prillinger’s^6^ findings, the structures of sensilla basiconica distinguished in the presently studied species belong to both ‘single-walled wall pore’ (SB2, SB3) and ‘double-walled wall pore’ classes (SB4).

Sensilla basiconica SB2 are present in some of the studied naucorids and are like other nepomorphans’ (Nepoidea, Ochteridea, Gelastocoridae and Aphelocheiridae)^33,34^. Sensilla basiconica SB3 probably derived from the type SB2, with a slight modification (they are longer and more acute distally) but are dominant in most species except *Cryphocricos montei*. The sensilla basiconica (SB3) of naucorids are similar to the ones of the mentioned nepomorphan taxa. Another type, sensillum SB4, is represented by grooved and porous sensilla. It was found in most naucorid species, Ochteridae and Gelastocoridae as the same type^34^ and in Corixoidea was identified as SB because, in that taxon, only one type, the olfactory sensilla, was distinguished^35^. Nevertheless, SB4 corresponds to the SBsh (sensilla basiconica short with longitudinal grooves and wall-pores) in pentatomid species^37^, in some species of Apoidea^43^ and in most other species of insects^44^.

Morphologically, chemosensitive sensilla basiconica are well represented in all the Naucoridae species studied, due to their surface being mostly covered with pores. The wall pores are essential for their olfactory function regardless of the shapes of the sensilla^5,6^. The present analysis of the different sensilla types suggests that, overall, the size and density of the sensilla are very changeable, disqualifying them from being a direct useful taxonomic tool. Still, the presence of the morphological types of sensilla is vital in comparisons among the species.

Specific features of some insects’ sensilla are proposed to be adaptations against water loss by evaporation. These sensilla belong to the "aporous" category into which thermo-hygroreceptive sensilla generally fall^11^. In the many studies of insects, the external structure of the sensilla ampullacea and coeloconica is compatible with the ultrastructural characters of thermo-hygroreception^6,45^. Hygroreceptors are associated in antagonistic pairs of a moist and a dry cell in the same sensilla with a thermoreceptor. In insect thermo-hygroreceptive sensilla all of the three cells respond to humidity changes, and two of the three cells react, in addition, to changes in temperature^46^.

In Naucoridae, sensilla ampullacea occur in six of the studied species (Table 2) on the third and fourth antennomeres. However, in some species, they could be hidden under a densely packed of mechanosensilla, making them hard to observe. Sensilla coeloconica were found in most species (Table 2), on the dorsal surfaces of the third and/or fourth antennomeres and on the tip of the last antennomere, except in *Cataractocoris marginiventris, Laccocoris* sp. and *Limnocoris asper*. Similarly, sensilla ampullacea and coeloconica have not been proven in all the studied species from previous studies on nepomorphans^24,33,34^.

The thermo-hygrosensilla of insects may be involved in mate-seeking or, in specialized cases, host-seeking, and such sensilla occur in virtually all insects where they have been looked for^11,45,47^. Although some authors suggest these sensilla to be redundant for organisms living in water^12^, other studies on water insects^33–35,41^ prove the existence of thermo-hygroreceptors, which may be relevant e.g. during the migrations and finding of new water bodies. The function of these sensilla in insects, including water insects, may be a crucial one because "For animals as small as insects, sunlit biotopes may be quite unmanageable if not quickly lethal in the absence of instant clues about their temperature and humidity"^45^.

The water habitats of naucorids differ, and the influence of insolation on the water temperature is also significant. The aquatic true bugs (Nepomorpha) usually possess thermo-hygrosensilla on the antennae^24,33,34^ as a remnant of land ancestors. However, they can be used to assess water temperature and habitats’ humidity.

#### Homology of sensilla in Naucoridae and Aphelocheiridae

We already know that the short rostrum and antennae of Naucoridae differentiate it from Aphelocheiridae, which has long antennae and rostrum^13,23,48,49^. The distinctive feature of Aphelocheiridae (*A. aestivalis* Fabricius, 1803), which live in running waters, is mainly a small number of mechanosensilla (ST2) on the 3rd antennomere and their absence on the first two^34^. The singular sensilla trichodea ST2 in Aphelocheiridae corresponds to numerous ST2 observed in all subfamilies of Naucoridae. Because the ST2 is common in other studied families of Nepomorpha^33,35^, other heteropteran insects^39,50,51^ and in insects in general^2^, the sensillum represents a plesiomorphic character, like the other mechanosensilla ST1, SCa (stress receptors) and SB1 (proprioceptor). The mechanosensilla ST3, ST4 and ST5 represent new state characters (an apomorphy) of the sensilla trichoidea in most studied species of Naucoridae, in contrast to other nepomorphans previously studied. So far, among the studied nepomorphan taxa, an olfactory plate-like sensillum was found only in Aphelocheiridae's species^34^, indicating an autapomorphy.

The two types of olfactory sensilla basiconica SB2 and SB3 found in Aphelocheiridae resemble the SB2 and SB3 described in this study. However, in Aphelocheiridae, several SB3 sensilla occur only on the antennae's tip, but it covered the third and fourth antennomeres in all species of naucorids, except in *Cryphocricos montei*. These sensilla basiconica represent plesiomorphic characters found in other nepomorphans^33,35^ and other insects^39,51,52^. Nonetheless, SB4 is absent in Aphelocheiridae, while it is widespread in Naucoridae, Ochteridae and Gelastocoridae. Generally, the SB4 (porous groove surface) type is found in most insects^53^.

We have discussed some sensillar characters of Naucoridae and Aphelocheiridae because both families' systematic positions are still unclear regarding these taxa's placement in the superfamilies. Some authors placed Aphelocheiridae together with Naucoridae in the superfamily Naucoroidea^23,25–29^, while others claim that Aphelocheiridae and Naucoridae belong to separate superfamilies, Aphelocheiroidea and Naucoroidea^26,31,32^.

No synapomorphy was found in the present analysis of the sensilla for Naucoridae and Aphelocheiridae. However, the subfamilies of Naucoridae also did not display a new set of sensilla common for all the subfamilies. The results suggest very variable traits of sensilla, and this may possibly be related to the influence of habitat pressure on the adaptive diversity of sensilla.

Some resemblance to Aphelocheiridae, in the distribution of antennal sensilla, could only be seen in a few species of Naucoridae (*Asthenocoris australis, Cryphocricos montei, Limnocoris asper* and *Limnocoris pusillus*), which are found in streams. The sensilla are not numerous in the mentioned species and form small groups on the third and fourth antennomeres. It might prove the hypothesis of the number of sensilla being reduced due to a preference for running water habitats. However, *Heleocoris,* which also prefers streams, and *Limnocoris volxemi*, do not display the reduction of antennal sensilla. Therefore, more material from other naucorids living in running water is needed to confirm or deny this hypothesis.

Probably, the morphology and abundance of antennal sensilla may vary depending on the insect’s family/subfamily/population. The types and sizes of sensilla may also vary depending on the geographical distribution of insects. For example, different patterns of trichoid and basiconic sensilla numbers were described in different populations of *Rhodnius prolixus* (Stål, 1859) (Heteroptera, Reduviidae, Triatominae) sampled from the east and the west of the Andes Mountains^54,55^. These differences suggest that the populations' geographical isolation was associated with the numbers of antennal sensilla^55^. So, these morphological differences in the sensorial organs in some insects might represent phenotypic plasticity that could result from different environmental conditions of the collected species, from different geographical populations^56^. The reason for that is that genetically similar organisms may have different phenotypes depending on environmental variables^57^.

## Conclusions

Insects’ adaptations to different conditions provide a plethora of fascinating examples of general morphology variations, including peripheral sensory organs. The development of sensitive peripheral detection systems seems to be important for adaptation to unique habitats. The family Naucoridae displays a great diversity in terms of antennae morphology and distribution of different antennal sensilla types. Moreover, the high level of the antennae's morphological diversity was visible among subfamilies and between species of the same subfamilies.

The study revealed new types of trichoid mechanosensilla (ST3, ST4, and ST5), with different distributions on the antennomeres of the studied Naucoridae species, when compared to other nepomorphans (Nepoidea, Ochteroidea, Aphelocheiroidea). The olfactory sensilla basiconica, distinguished in the present study, belong to both 'single-walled wall pore' (SB2, SB3) and 'double-walled wall pore' types (SB4). According to subfamilies/species, olfactory sensilla basiconica (SB2, SB3, SB4) have different configurations and abundance on the antennomeres. In the case of the naucorid species, these morphological differences in the sensorial organs (ST3, ST4, ST5, SB3) might represent phenotypic plasticity that could result from different environmental conditions of the species collected from different geographical populations. The basal set of sensilla (ST1, ST2, SCa, SB1, SA), included in the so-called conservative sensilla type, is similar in the studied naucorid species. No sensillar synapomorphy has been found between Aphelocheiridae and Naucoridae.

Overall, our results show that the sensillar equipment on the antennae of Naucoridae has some similarities with those described for previously studied species in other nepomorphan families and some similarities with terrestrial species of heteropterans and other insects.
